# Left colectomy with intracoporeal anastomosis: technical aspects

**DOI:** 10.1590/S1679-45082014MD3030

**Published:** 2014

**Authors:** Sérgio Eduardo Alonso Araujo, Victor Edmond Seid, Sidney Klajner, Alexandre Bruno Bertoncini

**Affiliations:** 1 Hospital Israelita Albert Einstein, São Paulo, SP, Brazil.; 2 Instituto do Câncer do Estado de São Paulo, São Paulo, SP, Brazil.

**Keywords:** Laparoscopy, Surgical procedures, operative, Colorectal surgery, Colorectal neoplasms, Intestinal polyps

## Abstract

Oncologic laparoscopic colectomy represents a fully validated surgical approach to the management of colorectal cancer. However, laparoscopic surgery for distal transverse and descending colon lesions remains a challenging procedure. A total laparoscopic approach to the left colectomy is an interesting option for critically ill patients although reports in the literature on this subject are scarce and its approach still not standardized because of its selective nature for indication. There are several advantages associated with conduction of totally laparoscopic approach to the left colon. Intracorporeal vessel sealing ensures an adequate lymph node dissection. Moreover, it enables the construction of a well-vascularized anastomosis. Ultimately, the occurrence of late wound complications are possibly reduced for the placement of a low abdominal incision exclusively used for specimen extraction. This paper aimed at describing our technique for a totally laparoscopic left colectomy for distal transverse and descending colon lesions.

## INTRODUCTION

Since the publication of the first laparoscopic colectomy its use has been increased.^(1)^ Moreover, randomized trials have demonstrated that laparoscopic surgery for colon cancer achieves good short-term and oncologic outcomes similar to those find in open surgery.^(2,3)^

However, laparoscopic surgery for transverse and descending colon cancer requires an advanced technique. Hence, only recently, studies have demonstrated the feasibility and safety of the laparoscopic resection for lesions located in the distal transverse and descending colon.^(4,5)^

Regarding the extent of lymphadenectomy, there is evidence in favor of extended right colectomies over segmental left colectomies for the treatment of distal transverse and left colon cancers.^(6)^ In addition, the extensive lymphadenectomy associated with the extended right colectomy for these tumors abolishes the risk of metachronous cancer.^(7)^ Moreover, the ileocolic anastomosis is probably safer than the colo-colic anastomosis. The relatively poor vascularization status in the distal transverse colon (the Griffiths’ point) is believed to add an increased risk of anastomotic complications.^(8)^

Incisional hernias after open surgery occur in 12 to 20% and may lead to significant morbidity. Midline extraction sites have a higher chance of hernias than non-midline.^(9)^ Therefore, a totally laparoscopic operation for distal may additionally result in less incisional hernias since the specimen extraction may be done through a small suprapubic incision.

Although advantages associated with totally laparoscopic approach to left colon lesions may be easily depicted, this operation represents a challenging procedure and for this reason, it is still not widespread and validated. This study aimed to report a standardized technical approach for totally laparoscopic left colectomy.

### Surgical technique

The patient is settled in the Lloyd-Davies position with the right arm along the body. The surgeon stands at the patient’s right, and the camera holder between patient’s legs. The assistant surgeon stands at the left side. During the procedure, the table is placed in moderate reverse Trendelemburg’s and tilted toward the right.

A 12mmHg-pneumoperitoneum is fashioned through a closed transumbilical technique (Verres needle). Five trocars are placed: a 10mmHg-trocar at the umbilicus, a 12mmHg-trocar at the right lower quadrant, and one 5mm-trocar is placed at each one of the other three abdominal quadrants.

**Figure 1:**
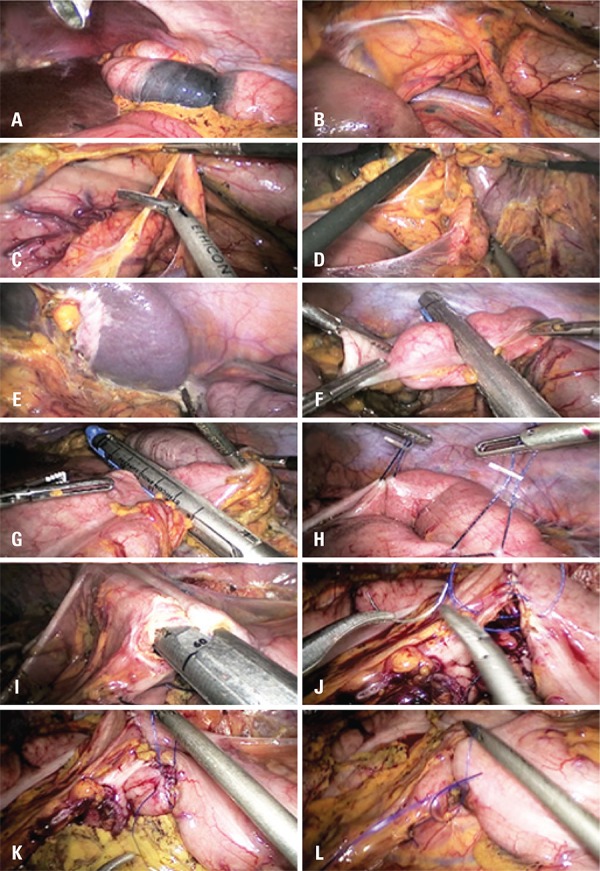
Totally laparoscopic left colectomy: steps proposed for standardization. (A) Lesion in the distal transverse colon (endoscopic tattoo mark). (B) Cranial folding of the distal transverse colon over the stomach (medial to lateral approach). (C) Division of the ion of the distal transverse colon on the ventral pancreatic aspect. (D) Blunt separation of the pancreatic tale from the dorsal aspect of the distal transverse colon. (E) Division of colonic retroperitoneal attachments at the splenic flexure level. (F) Intracorporeal transection of the colon at the level of the descending/sigmoid junction. (G) Intracorporeal transection of the distal transverse colon. (H) Apposition of the distal transverse colon to the sigmoid colon (isoperistaltic manner). (I) Anastomosis. (J) Colotomy closure (first suture plane). (K) Colotomy closure (second suture plane). (L) Closure of the mesenteric gap

At diagnostic laparoscopy, the left branch of the middle colic artery (MCA), the inferior mesenteric vein (IMV) and the left colic artery (LCA) must be identified along with the exact tumor location. Tumor location may be accomplished through preoperative endoscopic tattooing (Figure 1A). After complete transection of the round and falciform ligaments to the diaphragm level, the distal transverse colon is folded cranially over the stomach (Figure 1B). This presentation enables an adequate medial to lateral approach to structures at the Treitz angle (IMV, ventral pancreatic border and MCA). The peritoneal layer medial to the IMV is incised parallel to the vessel. Depending on the extent of resection, the surgeon decides whether to seal the IMV at its inferior pancreatic border. Therefore, when the sigmoid colon will not be resected, it is decided to seal and divide only the IMV branches using Harmonic Ace™ (Ethicon Endo-Surgery, Cincinnati, OH, USA). After, a dissection is conduced to separate the descending mesocolon of the Gerota’s plan from the medial aspect to the peritoneal lining to the left parietal gutter.

Subsequently, dissection and sealing of the left branch of the MCA are performed close to the mesenteric border of the transverse colon using a Harmonic Ace™. The peritoneal layer ventral to the body and tail of the pancreas is incised. This incision allows safe entry into the epiploon retrocavity. The insertion of distal transverse mesocolon to the pancreas is completely divided from the level of the left branch of the MCA to the splenic flexure (Figures 1C, 1D, and 1E). Next, the greater omentum is separated from the gastric curvature. Then, the mobilization of splenic flexure is completed by a latero-medial approach at the line of Toldt level.

If the sigmoid is to be preserved, the LCA must be identified from its origin at the IMA to the mesenteric border of the descending colon and left untouched. The intracorporeal transections of transverse (Figure 1F) and descending colon (Figure 1G) are accomplished using 3.5mm blue-load linear endoscopic staplers (Echelon Flex™ 60, Ethicon Endo-Surgery). The specimen, completely separated from all attachments, is then kept aside in the abdominal cavity.

The transverse and the left colon are then lined up (Figure 1H) side to side (isoperistaltic manner), with the help of two intracorporeal 20 cotton stiches. Two colostomies are made at the antimesenteric sides. A side-to-side colo-colic anastomosis is conducted with one fire of a 60mm blue endostapler load (Figure 1L). The enterotomy is then closed using a 3-0 PDS double layer running suture (Figures 1J and 1K). The mesenteric gap should be carefully closed using interrupted 3-0 cotton stitches (Figure 1L).

The specimen is retrieved and properly protected through a small suprapubic incision. In general, drains are not used.

## DISCUSSION

The best surgical option for minimally invasive treatment of distal transverse and descending colon tumors remains controversial. Therefore, different approaches have been proposed. The main variables brought into the equation remain the extent of lymphadenectomy and anastomotic safety. The most frequently proposed operations are the left segmental colectomy and the right extended colon resection.^(10)^ Regardless of the technique, adherence to oncologic parameters is mandatory. The main advantages associated with the extended right colectomy as opposed to the left segmental colectomy are the greater extent of lymphadenectomy and safety of ileocolic anastomosis associated to the first surgery. However, an extended surgery may be associated with increased morbidity and eventually unnecessary in patients with complete endoscopic examination of the colon and harboring early tumors. In such situation, totally laparoscopic left colectomy is a suitable indication.

During the totally laparoscopic left colectomy, adequate lymphadenectomy derives from intracorporeal vascular control. Moreover, an intracorporeal anastomosis minimizes the risk of bowel twisting, and through avoiding exteriorization of the stumps, it may reduce bowel traction, which can affect anastomotic irrigation, especially in obese patients. Ultimately, a superior cosmetic effect and less incisional complications can be associated to a suprapubic incision used to specimen retrieval.

## CONCLUSION

Although some advantages associated to the procedure seem speculative, the totally laparoscopic left colectomy represents a reproducible procedure. However, the professional conducting the procedure must have adequate laparoscopic skills. Because this approach has a selective indication, technical standardization is demanded, and future trials should be encouraged.
