# Supplementary material for: Left colectomy with intracoporeal anastomosis: technical aspects
Source: Einstein (Sao Paulo). 2014 Jul-Sep;12(3):386–8. doi: 10.1590/S1679-45082014MD3030 (PMC4872958; doi:10.1590/S1679-45082014MD3030)
Supplement: Translation

# Translation: Colectomia esquerda com anastomose intracorpórea: aspectos técnicos

**Authors:** Sérgio Eduardo Alonso Araujo, Victor Edmond Seid, Sidney Klajner, Alexandre Bruno Bertoncini

**Affiliations:** 1 Hospital Israelita Albert Einstein, São Paulo, SP, Brasil.; 2 Instituto do Câncer do Estado de São Paulo, São Paulo, SP, Brasil.

**Keywords:** Laparoscopia, Procedimentos cirúrgicos operatórios, Cirurgia colorretal, Neoplasias colorretais, Pólipos intestinais

## Abstract

A colectomia oncológica por videolaparoscopia representa uma abordagem cirúrgica validada no tratamento radical do câncer colorretal. No entanto, a cirurgia laparoscópica para lesões de cólon transverso e descendente distal persiste como um procedimento tecnicamente complexo. A abordagem totalmente laparoscópica para a realização da colectomia esquerda é uma opção interessante para pacientes selecionados, ainda que parcialmente avaliada na literatura e, sobretudo, carecendo de padronização técnica, devido à natureza seletiva de sua indicação. Existem várias vantagens associadas à realização da colectomia esquerda totalmente laparoscópica. A realização do controle vascular por videolaparoscopia assegura uma adequada extensão da dissecção linfonodal. Além disso, permite a construção de uma anastomose bem vascularizada. Finalmente, a ocorrência de complicações tardias da ferida pode ser reduzida devido ao emprego de uma incisão abdominal utilizada exclusivamente para a retirada do amostra cirúrgica. Este artigo teve como objetivo descrever uma proposta técnica de padronização da colectomia esquerda totalmente laparoscópica para lesões de cólon transverso e descendente distal.

## INTRODUÇÃO

Desde a publicação da primeira colectomia laparoscópica, o uso desse procedimento tem aumentado.^(1)^ Além disso, ensaios clínicos randomizados têm demonstrado que a cirurgia laparoscópica para câncer de cólon alcançou bons resultados em curto prazo, além de desfechos oncológicos similares aos da cirurgia aberta.^(2, 3)^ No entanto, a cirurgia laparoscópica para tumores de cólon transverso e descendente demanda uma técnica avançada. Apenas recentemente, estudos demonstraram a facilidade e segurança da ressecção laparoscópica para lesões localizadas no cólon transverso e descendente distal.^(4, 5)^

Em relação à extensão da linfadenectomia, há evidência em favor das colectomias direitas estendidas em sobreposição às colectomias do segmento esquerdo para tratamento de câncer de cólon transverso distal e esquerdo.^(6)^ Além disso, a linfadenectomia estendida associada à colectomia direita estendida, para esses tumores, elimina o risco de carcinoma metacrônico.^(7)^ A anastomose ileocólica é provavelmente mais segura se comparada à anastomose colocólica. Acredita-se que o estado de vascularização deficiente no cólon transverso distal (ponto de Griffiths) represente aumento do risco de complicações anastomóticas.^(8)^

As hérnias incisionais após cirurgia aberta ocorrem em 12 a 20% dos casos e podem levar à morbidade significante. Os locais de extração transversal têm maior chance de ocorrência de hérnias do que as não transversais.^(9)^ Desse modo, a operação totalmente laparoscópica distal pode resultar em menor incidência de hérnias incisionais, desde que a extração de amostras possa ser feita por meio de uma pequena incisão suprapúbica.

Apesar de vantagens associadas à abordagem totalmente laparoscópica em lesões do cólon esquerdo poderem ser facilmente descritas, esse procedimento representa desafio e, por esse motivo, ainda não é difundido e nem validado. Nosso objetivo foi relatar uma abordagem técnica padronizada para colectomia esquerda totalmente laparoscópica.

### Técnica cirúrgica

O paciente fica na posição de Lloyd-Davies, com o braço direito ao longo do corpo. O cirurgião permanece do lado direito e seu assistente do lado esquerda da mesa cirúrgica. A câmera é posicionada entre as pernas do paciente. Durante o procedimento, a mesa cirúrgica está em posição de Trendelemburg reverso-moderada e inclinada para direita.

Um pneumoperitônio de 12mmHg é conduzida por meio de incisão transumbilical fechada (agulha de Verres). Cinco trocartes são posicionados: um trocarte de 10mmHg no umbigo, um trocarte de 12mmHg no quadrante inferior direito, e um trocarte de 5mmHg em cada um dos outros três quadrantes abdominais.

No diagnóstico laparoscópico, o ramo esquerdo da artéria cólica média (ACM), a veia mesentérica inferior (VMI) e artéria cólica esquerda (ACE) devem ser identificadas juntamente da localização exata do tumor. A localização do tumor pode ser marcada utilizando-se tatuagem endoscópica pré-operatória (Figura 1A). Após a transecção completa dos ligamentos circulares e falciformes e dos ligamentos falciformes ao nível do diafragma, o cólon transverso distal é dobrado cranialmente sobre o estômago (Figura 1B). Essa apresentação possibilita uma abordagem medial a lateral adequada para estruturas no ângulo de Treitz (VMI, borda pancreática ventral e ACM). A camada peritoneal medial à VMI é removida paralelamente aos vasos. Dependendo da extensão da ressecção, o cirurgião decide se sela ou não a VMI em sua borda pancreática inferior. Desse modo, quando o colón de sigmoide não for ressecado, decide-se pela selagem e dividem-se apenas os ramos da VMI utilizando *Harmonic Ace* ^®^ (*Ethicon Endo-Surgery, Cincinat* *ti, OH, EUA*). Posteriormente, realiza-se dissecção para separar o mesocólon descendente do plano de Gerota do aspecto medial, para revestimento peritoneal para a abertura parietal esquerda.

**Figura 1:** Colectomia total laparoscópica esquerda: passos propostos para padronização. (A) Lesão em cólon transverso distal (marcação com tatuagem endoscópica). (B) Dobragem cranial de cólon transverso distal sobre o estômago (abordagem medial e lateral). (C) Divisão de íons do cólon transverso distal no aspecto pancreático ventral. (D) Separação da cauda pancreática do aspecto dorsal do cólon transverso distal. (E) Divisão de ligamentos colônicos retroperitoneal no nível da flexura esplênica. (F) Transecção intracorpórea do cólon no nível da junção descendente/sigmoide. (G) Transecção intracorpórea do cólon transverso distal. (H) Aposição do cólon transverso distal para cólon sigmoide (modo isoperistáltico). (I) Anastomose. (J). Fechamento de colostomia (primeiro plano da sutura). (K) Fechamento de colostomia (segundo plano da sutura). (L) Fechamento da abertura mesentérica

Após, procedem-se à dissecção e à selagem do ramo esquerdo do ACM com Harmonic Ace^®^ próximo da borda mesentérica do cólon transverso. A camada peritoneal ventral do corpo e da cauda do pâncreas é removida. Tal procedimento possibilita entrada segura na retrocavidade dos epiplons. A inserção do mesocólon transverso distal ao pâncreas é completamente divida, a partir do nível do ramo esquerdo da ACM até a flexura esplênica (Figura 1C, 1D e 1E). A seguir, o omento maior é separado da curvatura gástrica. Então, a mobilização da flexura explênica é completada por meio de abordagem lateromedial no nível da linha de Toldt.

Se o sigmoide deve ser preservado, a ACE deve ser identificada a partir de sua origem na VMI até a borda mesentérica do cólon descendente e esquerdo preservado. As transecções intracorpóreas do cólon transverso (Figura 1F) e descendente (Figura 1F) são realizadas utilizando grampeador linear endoscópico de 3,5mm carga azul (*Echelon Flex* ^*®*^ *60*, *Ethicon Endo* *-Surgery*). A amostra, completamente separada de todos os ligamentos, é, então, mantida em reserva na cavidade abdominal.

O cólon transverso e esquerdo são, dessa maneira, alinhados (Figura 1H) lado a lado (modo isoperistáltico), com ajuda de duas suturas intracorpóreas com fio de algodão 20. Duas colostomias são realizadas nos lados antimesentéricos. Uma anastomose colocólica em plano único é realizada com grampeador linear endoscópico de 60mm carga azul (Figura 1L). A enterotomia é então fechada com sutura em camada dupla 3-0 PDS (Figuras 1J e 1K). A abertura mesentérica deve ser cuidadosamente fechada utilizando sutura contínua com fio de algodão 3-0 (Figura 1L)

A amostra é recuperada e apropriadamente protegida por meio de pequena incisão suprapúbica. Geralmente, drenos não são utilizados.

## DISCUSSÃO

A melhor opção cirúrgica para tratamento minimamente invasivo de tumores do cólon transverso e descendente distal é controversa. Contudo, diferentes abordagens têm sido propostas. As principais variáveis posicionadas na equação permanecem a extensão da linfadenectomia e a segurança da anastomose. Os procedimentos mais frequentemente propostos são a colectomia segmentar esquerda e a ressecção do cólon direito estendido. pe^(10)^ Independentemente da técnica adotada, a adesão aos parâmetros oncológicos é primordial. As principais vantagens associadas a colectomia direita estendida, em oposição à colectomia do segmento esquerdo, são a maior extensão da linfadenectomia e a segurança da anastomose ileocólica, associadas à primeira cirurgia. Porém, a operação estendida pode estar associada ao aumento de morbidade e eventualmente ser desnecessária em pacientes com exame endoscópico completo do cólon e alojamento prévio de tumores. Em tal situação, uma colectomia total laparoscópica esquerda é a indicação mais adequada.

Durante a colectomia total laparoscópica esquerda, a linfadenectomia adequada deriva do controle vascular intracorpóreo. Além disso, uma anastomose intracorpórea minimiza o risco de obstrução intestinal e, por evitar a exteriorização dos cotos, ela pode reduzir a tração intestinal que, por sua vez, pode afetar a vascularização da anastomose, principalmente em pacientes obesos. Por fim, um melhor efeito cosmético e menos complicações podem ser associados à incisão suprapúbica utilizada na recuperação da amostra.

## CONCLUSÃO

Apesar de algumas vantagens associadas ao procedimento parecerem especulativas, a colectomia laparoscópica esquerda total representa um procedimento reproduzível. Porém, o profissional que conduz o procedimento deve possuir habilidades laparoscópicas adequadas. Devido à indicação dessa abordagem ser seletiva, é necessária sua padronização técnica e futuros ensaios clínicos devem ser incentivados.
